# The effects of sleep patterns and chronotype on depressive symptoms in adolescents: a cross-sectional study using data from the 19th Korea Youth Risk Behavior Web-based Survey

**DOI:** 10.7586/jkbns.25.043

**Published:** 2025-11-26

**Authors:** Su-Jeong Jeong, Chul-Gyu Kim, Jung-Ha Son

**Affiliations:** 1 College of Nursing, Chungbuk Health & Science University, Cheongju, Korea; 2 College of Nursing, Chungbuk National University, Cheongju, Korea; 3 Chungbuk Emergency Medical Support Center, National Emergency Medical Center, National Medical Center, Cheongju, Korea

**Keywords:** Adolescent, Chronotype, Depression, Sleep

## Abstract

**Purpose:**

This secondary data analysis examined the relationship between sleep patterns (including bedtime and wake-up time), chronotype, and depressive symptoms in adolescents aged 13~18 years.

**Methods:**

Data were derived from the 19th Youth Risk Behavior Survey conducted in 2023. Of the 52,880 respondents, 5,893 were excluded, resulting in a final analytic sample of 46,987 adolescents. Complex sample logistic regression analyses were conducted using SAS version 9.4 to investigate the effects of sleep patterns and chronotype on depressive symptoms.

**Results:**

Among the 46,987 participants, 11,967 reported depressive symptoms, yielding an estimated prevalence of 25.3%. Statistically significant differences in bedtime, wake-up time, sleep duration, and chronotype were identified between adolescents with and without depressive symptoms (*p* < .001). The mid-sleep time on free days corrected for sleep debt on school days was significantly later among those with depressive symptoms (5.49 ± 0.03) compared with those without (5.36 ± 0.02; *p* < .001). Significant correlates of depressive symptoms included sex, living with family, household income, school grade, current drinking and smoking, habitual drug use, perceived health status, anxiety, stress awareness, loneliness, and sleep duration. Each additional hour of sleep was associated with a 0.98-fold lower risk of depressive symptoms (95% confidence interval = 0.96~0.99, *p* = .021).

**Conclusion:**

This study demonstrated that sleep duration, sleep patterns, and chronotype are associated with depressive symptoms in adolescents. Interventions should target extending sleep duration through earlier bedtimes and encouraging a shift from evening-type to morning-type chronotypes, which may help alleviate depressive symptoms.

## INTRODUCTION

The prevalence of depressive symptoms among Korean adolescents increased for both males (from 22.4% in 2021 to 24.2% in 2022) and females (from 31.4% in 2021 to 33.5% in 2022) [1]. Considering that depressive symptoms are a major psychological risk factor leading to suicide [2], it is essential to identify the factors contributing to elevated levels of depressive symptoms in adolescents in order to reduce the risk of suicide among this population.

During adolescence, individuals experience significant changes in social development and physical growth, leading to difficulties in emotional regulation and psychological distress [2]. Various personal, familial, and social factors contribute to depressive symptoms. High academic stress and low academic achievement in adolescents are associated with increased depression. Intimacy and attachment with peers, as well as maladaptive relationships with teachers, are also closely linked to depression [3]. Anxiety, another emotional difficulty faced by adolescents, is closely related to depression; it not only manifests, in severe cases, as problems such as interpersonal difficulties, delinquency, self-harm, and suicide [2], but also has a direct positive effect on depression, indicating that higher levels of anxiety are associated with more severe depression [4]. In addition, higher levels of stress perception and greater experiences of loneliness are associated with more severe depressive symptoms, suggesting that adolescents who feel more stressed or isolated are at increased risk of depression [5]. In particular, Korean adolescents experience considerable psychological difficulties due to the university entrance exam–centered education system and the stress associated with college admissions. This situation is well reflected in the results of the 2020 Korean Youth Risk Behavior Web-based Survey (KYRBS), in which 34.2% of adolescents reported stress perception, 25.2% experienced depressive symptoms, and 10.9% had suicidal ideation [6]. KYRBS has been conducted annually since 2005 to assess the health behaviors of adolescents in Korea and to produce national health and health-related indicators. The survey targets students from the first year of middle school through the third year of high school nationwide, and covers a total of 106 items, including smoking, alcohol consumption, physical activity, and mental health [7].

Previous studies have shown that sleep patterns and quality are related to adolescent depression. A study of Australian adolescents reported that short sleep duration and poor subjective sleep quality mediated the relationship between age and depressive symptoms, suggesting that sleep-related developmental changes contribute to increased depression during adolescence [8]. Delayed sleep onset has also been identified as a risk factor for depressive symptoms in young people aged 12~25 years [9]. In Korean adolescents, these associations are particularly concerning given the intense academic environment. Supporting this, Shin [10] reported that inadequate sleep duration was significantly associated with depressive symptoms.

Delayed sleep onset, or late bedtime, is associated with an evening chronotype, which is a personal characteristic [11]. Chronotype refers to interindividual differences in sleep-circadian rhythms, specifically the sleep–wake cycle and daily activities. It distinguishes whether individuals prefer to be awake or active in the morning or evening and classifies them as either falling under the categories of morningness or eveningness [12]. Morning-type individuals tend to go to bed and wake up early, demonstrating better performance during the early hours of the day, whereas evening-type individuals have later bedtimes and wake-up times, exhibiting better performance in the late afternoon or evening [13]. The higher levels of depression observed in individuals with evening chronotypes may be attributed to their low sensitivity in their behavioral activation system, leading to diminished reward responsiveness and positive emotional levels, thus contributing to depressive symptoms [14]. Furthermore, one’s circadian rhythm is associated with the secretion of serotonin, norepinephrine, dopamine, and melatonin, all of which influence mood regulation [15]. Circadian disruptions, delayed sleep onset, and short sleep duration are linked to abnormal patterns in the secretion of these hormones as well as cognitive and behavioral functions, contributing to the development of depressive symptoms [12]. In particular, adolescence is a critical period characterized by a shift from morningness to eveningness [16]. Extreme evening-type characteristics during this phase may hinder the formation of healthy lifestyle habits and interfere with school-required activities and academic achievement, underscoring the need for appropriate interventions, especially in Korea’s education-intensive environment [17].

Prior research has shown associations between chronotype and depressive symptoms in adults, and between sleep patterns and depression in adolescents [8,18]. However, studies focusing on adolescent chronotypes—particularly the transition toward eveningness—are scarce, and existing findings are limited by small, localized samples. Given Korea’s unique academic pressures and lifestyle patterns, this study used nationally representative data from the 19th KYRBS to examine the relationship between sleep patterns, chronotype, and depressive symptoms in adolescents aged 13~18 years.

## METHODS

### 1. Study design

This study employed a cross-sectional descriptive design using secondary data analysis.

### 2. Participants

This study used raw data from the 19th KYRBS conducted in 2023, which included a total of 52,880 participants [7]. Among these participants, 5,893 individuals with missing data related to general characteristics, physical and mental health, health behaviors, and sleep were excluded from analysis, resulting in the analysis of data from 46,987 participants.

### 3. Instruments

#### 1) Sleep patterns and chronotype

Sleep patterns were defined in terms of bedtime, wake-up time, and sleep duration. KYRBS [7] assessed bedtime and wake-up time by asking participants, "What time (in hours and minutes) do you typically go to bed and wake up during the past 7 days (on weekdays and weekends)?" Sleep duration was calculated by subtracting the bedtime from the wake-up time [18].

Chronotype was assessed using the mid-sleep time on free days corrected for sleep debt (MSF_SC_) [19]. MSF_SC_ is calculated using the formula MSF_SC_ = MSF [weekend bedtime + (total weekend sleep duration ÷ 2)] – (0.5 × [total weekend sleep duration – (5 × total sleep duration during weekdays + 2 × total weekend sleep duration) ÷ 7] [20]. Chronotype was defined based on the midpoint of sleep duration on free days (mid-sleep on free days, MSF), reflecting an individual's most natural circadian rhythm without the interference of alarm clocks or school obligations [20]. Individuals with an evening chronotype, however, may attempt to compensate for sleep debt during the weekdays by sleeping less and then sleeping more on weekends, which can lead to an increased MSF value. To address this, a corrected measure was used: the mid-sleep time on free days corrected for sleep debt accumulated over school days (MSF_SC_). Higher MSF_SC_ values correspond to a later chronotype (eveningness), whereas lower values indicate an earlier chronotype (morningness) [19]. In this study, MSF_SC_ was used as a continuous variable to assess chronotype [7].

#### 2) Mental health variables

The mental health variables assessed were depressive symptoms, anxiety, stress, and loneliness. Depressive symptom was measured using a single-item question: "During the past 12 months, have you felt so sad or hopeless almost every day for two weeks or more in a row that you stopped doing some usual activities?" Responses were recorded as binary data with options of "yes" or "no." Participants who responded ‘yes’ were classified into the depressive symptom group, while those who responded ‘no’ were classified into the non-depressive symptom group [7,21]. Anxiety was assessed using a Korean-translated version of the Generalized Anxiety Disorder 7-item scale (GAD-7) [22]. The GAD-7 includes the question, "How often have you been bothered by the following problems?" followed by seven items such as "Feeling nervous, anxious, or on edge." Respondents rated their experience on a 4-point scale ranging from 0 for "Not at all bothered" to 3 for "Nearly every day." The total score ranged from 0 to 21 points, with higher scores indicating higher levels of anxiety. In this study, a cut-off score of 10 points, as suggested by Spitzer et al. [22], was used; individuals scoring 10 or above were considered to have anxiety. Stress was measured using the question, "How much stress do you usually feel?" with a total of five response options: "Very much" (5 points), "Much" (4 points), "A little" (3 points), "Not much" (2 points), and "Not at all" (1 point). Loneliness was assessed using the question, "How often have you felt lonely during the past 12 months?" using a five-option scale: "Always" (5 points), "Often" (4 points), "Sometimes" (3 points), "Rarely" (2 points), and "Not at all" (1 point).

#### 3) General characteristics

General characteristics assessed were age, gender, school year, household income, living with family, and academic performance. The school year was categorized into middle and high school. Household income was classified into three groups based on the individual's subjective economic status: high, middle (upper middle, middle, and lower middle), and low [23-25]. Living with family was determined by whether the adolescent lived with their family, with a "yes" answer indicating cohabitation with family and a "no" answer indicating either residence at relatives' homes, childcare facilities, boarding houses, independent living, or dormitories. Academic performance was evaluated based on grades over the past 12 months and categorized into three groups: high, middle (upper middle, middle, and lower middle), and low [21,24,25].

#### 4) Health status and behavior-related variables

Health status and behavior-related variables assessed were perceived health status, obesity, physical activity, current drinking and smoking, and habitual drug use. Perceived health status was assessed using the question, "How would you rate your health generally?" on a 5-point scale: "Very healthy" (5 points), "Healthy" (4 points), "Average" (3 points), "Not very healthy" (2 points), and "Very unhealthy" (1 point). Obesity was classified using body mass index (kg/m²), calculated from self-reported height and weight data obtained from the KYRBS, according to the 2017 Korean National Growth Chart for children and adolescents, which categorizes individuals as underweight, normal weight, or obese (including overweight and obesity) [21]. Physical activity was categorized into moderate- and vigorous-intensity activities [26]. Moderate physical activity was determined based on the criterion that if, in the past seven days, participants engaged in any physical activity that increased their heart rate or left them with shortness of breath for more than 60 minutes on at least five days, it was classified as practiced; otherwise, it was deemed not practiced. For vigorous physical activity, if participants engaged in high-intensity activities that caused significant shortness of breath or sweating on more than three days in the past seven days, it was classified as practiced; otherwise, it was considered not practiced. For current drinking, participants answered the question, "In the past 30 days, on how many days did you consume at least one drink of alcohol?" If they reported "none" for the past 30 days, their response was classified as "no," and if they indicated drinking on one or more days, their response was classified as "yes." For current smoking, participants responded to the question, "In the past 30 days, on how many days did you smoke even one cigarette?" If they indicated no smoking for the past 30 days, their response was classified as "no," whereas if they reported smoking on one or more days, their response was classified as "yes." Habitual drug use was assessed using the question, "Have you ever habitually or intentionally used drugs (such as tranquilizers, stimulants, sleeping pills, appetite suppressants, and narcotic analgesics) or inhaled substances like glue, marijuana, cocaine, or butane?" Respondents answered with "no" or "yes."

### 4. Data collection

The researcher obtained the raw data after receiving approval to access statistical information from the Korea Disease Control and Prevention Agency website and modified the dataset for analysis according to the study objectives. The target population of the 19th KYRBS comprised middle and high school students nationwide as of April 2023. The KYRBS is an annual self-administered online survey and a nationally approved statistical survey (approval number 117058). In accordance with the Enforcement Rule of the Bioethics and Safety Act, it is conducted without review by an Institutional Review Board.

Participants were selected through a multi-stage process of stratification, sample allocation, and sampling. In the stratification stage, the population was divided into 117 strata based on region and school level. During the sample allocation stage, 400 middle schools and 400 high schools were chosen. Sampling was then conducted using a stratified cluster sampling method, with schools serving as the first-stage sampling units and classes as the second-stage units. All students in the selected classes were included in the survey, except those with long-term absenteeism, special education needs, or literacy difficulties.

For the survey administration, the principal of each participating school designated a survey support teacher, excluding the homeroom teacher of the sample class. On the survey day, the support teacher guided students to an internet-enabled school computer lab, where each student was randomly assigned to a computer. Using their assigned participation numbers, students accessed the survey website and completed the questionnaire. The survey support teacher supervised the process according to the official guidelines, which prohibited the homeroom teacher from entering the room, forbade viewing of students’ computer screens, and restricted the provision of answers to survey questions. The entire survey was conducted during a single class period of 45~50 minutes [7].

### 5. Data analyses

Data analysis was conducted using SAS version 9.4 (SAS Institute Inc., Cary, NC, USA) for Windows. The KYRBS data were analyzed using complex sample methods accounting for the sample design characteristics in the raw data, including stratification variables, cluster variables, weights, and a finite population correction factor. To examine differences in depressive symptoms by participants' characteristics, complex sample t-tests and Rao–Scott chi-square tests for cross-tabulations were performed, all incorporating the sample design features.

To assess the influence of sleep patterns and chronotype on depressive symptoms status (yes/no), we conducted complex sample logistic regression. Variables were selected using the backward elimination method and results were reported as odds ratios (ORs) with 95% confidence intervals (CIs). The statistical significance level was set at *p* < .05.

### 6. Ethical considerations

This study was conducted with the approval of the Institutional Review Board of Asan Medical Center (Approval Number: 2024-1117).

## RESULTS

### 1. Participants' levels of depressive symptoms, sleep patterns, and chronotype

The number of individuals exhibiting depressive symptoms was 11,967 out of 46,987 participants, representing 25.3%. The most common bedtime was between 01:00 and 01:59, reported by 27.9% of participants. Overall, 70.1% of participants reported going to bed between 00:00 and 02:59. The most common wake-up time was between 07:00 and 07:59, experienced by 61.2% of participants. In total, 88.3% of participants woke up between 06:00 and 07:59. Average sleep duration on weekdays was 6.24 ± 0.01 hours. On weekdays, 25.9% of participants reported sleeping six to seven hours, followed by 24.3% who slept five to six hours and 19.9% who slept seven to eight hours. Overall, 70.1% of participants reported a sleep duration of five to eight hours. The chronotype score (MSF_SC_) was measured at 5.39 ± 0.01 (Table 1).

### 2. Differences in depressive symptoms according to participant characteristics

Statistically significant differences were observed between participants with and without depressive symptoms in sex, living with family, household income, academic performance, current drinking, current smoking, habitual drug use, and perceived health status (*p* < .001, Table 2). Participants with depressive symptoms included a higher proportion of women, lower academic performance, more individuals with low household income levels, and a lower proportion living with family compared with those without depressive symptoms (*p* < .001). The current drinking rate was 15.3% among participants with depressive symptoms, higher than 8.9% among those without depressive symptoms (*χ*^2^ = 289.93, *p* < .001). Similarly, the current smoking rate was 6.8% versus 2.8% (*χ*^2^ = 259.46, *p* < .001), and 3.2% reported habitual drug use compared with 0.7% among those without depressive symptoms (*χ*^2^ = 261.73, *p* < .001). Perceived health status was lower among participants with depressive symptoms (mean 3.49 ± 0.01) than those without depressive symptoms (3.84 ± 0.01; t = 31.81, *p* < .001). Awareness of stress was higher (3.79 ± 0.01 vs. 3.07 ± 0.01; t = −70.80, *p* < .001), as was the experience of loneliness (3.31 ± 0.01 vs. 2.34 ± 0.01; t = −88.33, *p* < .001). In addition, 30.5% of participants with depressive symptoms reported experiencing anxiety, compared with 5.9% of those without depressive symptoms (*χ*^2^ = 6115.38, *p* < .001; Table 2).

### 3. Differences in depressive symptoms based on sleep patterns and chronotype

Significant differences were observed in sleep patterns and chronotype (MSF_SC_) between participants with and without depressive symptoms (*p* < .001). For bedtime, the proportion of participants with depressive symptoms was lowest (20.3%) among those who went to bed between 22:00 and 23:59. As bedtime was delayed beyond midnight, this proportion increased, reaching the highest level (35.2%) among those going to bed after 3:00 (*χ*^2^ = 388.93, *p* < .0001). For wake-up time, the lowest proportion with depressive symptoms (24.1%) was observed among those waking between 7:00 and 7:59, whereas the highest (36.5%) was found among those waking between 4:00 and 4:59 (*χ*^2^ = 73.30, *p* < .001). In terms of sleep duration, depressive symptoms were least common (19.5%) among those sleeping eight to ten hours, while the highest proportion (38.7%) was observed among those sleeping fewer than four hours (*χ*^2^ = 452.87, *p* < .001). The average sleep duration was significantly shorter for participants with depressive symptoms (5.59 ± 0.02 hours) than for those without (6.32 ± 0.01 hours; t = 21.11, *p* < .001). MSF_SC_ was also significantly later among participants with depressive symptoms (5.49 ± 0.03) compared with those without (5.36 ± 0.02; t = −4.72, *p* < .001; Table 3).

### 4. Factors influencing depressive symptoms

Sex, living with family, household income, academic performance, current drinking, current smoking, experience of habitual drug use, perceived health status, anxiety, awareness of stress, experience of loneliness, and sleep duration were significantly associated with depressive symptoms (*p* < 0.05). Female students had significantly higher odds of depressive symptoms compared to male students (OR = 1.15, 95% CI = 1.09~1.22). Those not living with family had an OR of 1.19 (95% CI = 1.05~1.35) for depressive symptoms; middle academic performance OR was 1.30 (95% CI = 1.20~1.41), and that for low academic performance was 1.48 (95% CI = 1.31~1.67). Current drinking had an OR of 1.35 (95% CI = 1.24~1.48) for depressive symptoms; current smoking OR was 1.72 (95% CI = 1.50~1.96), that for experience of habitual drug use was 1.98 (95% CI = 1.57~2.49), and that for anxiety was 2.28 (95% CI = 2.11~2.46).

Each 1-point increase in awareness of stress levels raised depressive symptom risk by 1.72 times (95% CI = 1.66~1.79), and for each increase of 1 point in experience of loneliness scores, the risk also increased by 2.00 times (95% CI = 1.94~2.07). Meanwhile, the OR for middle household income was protective at 0.81 (95% CI = 0.74~0.89). Perceived health status and sleep duration were also protective factors. Each 1-point increase in perceived health status decreased depressive symptom risk by 0.96 times (95% CI = 0.93~0.99), and for each increase of one hour of sleep, the risk also decreased by 0.98 times (95% CI = 0.96~0.99; Table 4).

## DISCUSSION

This study aimed to explore the relationship between sleep patterns, chronotype, and depressive symptoms in Korean adolescents, based on self-reported data from the KYRBS. Among the 46,987 participants, 11,967 (25.3%) reported experiencing depressive symptoms. While this figure is slightly lower than the adolescent depression experience rate of 29.3% reported in previous studies in 2022 [23], it is significantly higher than the 7.3% observed in a 2023 survey targeting adults aged 19 and older [27]. The decrease from 29.3% to 25.3% contrasts with reports showing increased depression rates among adults following the coronavirus disease 2019 (COVID-19) pandemic [27]. A possible explanation is the end of the pandemic in 2023, which normalized school life and reduced factors associated with depressive symptoms, such as anxiety, stress, alcohol consumption, and smoking [28]. However, the adolescent depression experience rate was reported as 27.7% in 2024 [28], indicating that the prevalence of depressive symptoms among adolescents remains a serious concern, affecting approximately one in four youths. Identifying the multifactorial determinants of adolescent depression is therefore critical to inform early screening, guide targeted interventions, and support the development of school- and community-based preventive programs.

In this study, several general characteristics, physical health status, health behaviors, and mental health-related variables were significantly associated with depressive symptoms in adolescents, consistent with previous findings [5,10,23–25,29]. Importantly, sleep-related variables—including bedtime, wake-up time, sleep duration, and chronotype—also showed associations with depression [5,10,24]. The average weekday sleep duration was 6.24 ± 0.01 hours, with 50.2% (n = 23,042) of participants reporting five to seven hours of sleep. Only 13.4% achieved the recommended eight to ten hours advised by the American Sleep Foundation [30], indicating widespread insufficient sleep. This pervasive sleep deprivation likely reflects Korea’s intense academic culture, characterized by extensive after-school private tutoring and high smartphone use before bedtime [31,32].

The findings also show that the percentage of depressive symptoms was markedly higher among those who slept for less than five hours. This is consistent with previous research indicating that middle school students with sleep durations of less than five hours have an OR of 2.78 for depression, while high school students have an OR of 1.98 times for depression [10]. This study found that going to bed after 2:00 and waking before 6:00 were significantly associated with a higher percentage of depression symptoms. These results are consistent with those of studies conducted in South Korean adults, which found that going to bed after 1:00 was associated with a higher percentage of depression [11], and similar findings were observed in research involving Japanese adults, where late bedtimes increased the risk of depressive symptoms [33]. In this study, 55.8% of the participants went to bed after 1:00. Late bedtimes often lead to insufficient sleep, which not only results in depressive symptoms and anxiety but also impairs attention and learning abilities, potentially leading to problematic behaviors [34]. Given the association between self-reported sleep duration and depressive symptoms among Korean adolescents [10], strategies to improve sleep should be emphasized. In particular, extending average sleep duration may be facilitated through evidence-based school-based sleep education programs [35] and comprehensive sleep hygiene interventions. Such interventions address nutrition, emotional regulation, behavioral factors (e.g., limiting screen time or caffeine use), and environmental and temporal conditions that influence sleep [36]. In addition, excessive smartphone use before bedtime increases arousal and suppresses melatonin secretion owing to blue light exposure, making it difficult for the brain to enter sleep mode. Adolescents use their smartphones for approximately two hours before sleep; thus, smartphone usage should be reduced to increase nighttime sleep duration [31].

Chronotype, assessed using MSF_SC_, was 5.36 ± 0.02 among participants without depressive symptoms and 5.49 ± 0.03 among those with depressive symptoms, reflecting a later evening chronotype in the latter group. This finding aligns with results indicating that evening-type adolescents in South Korea tend to experience lower sleep quality and cumulative fatigue, which negatively impacts their physical functioning and makes them more vulnerable to depressive symptoms [24]. Other studies on Korean adolescents have also demonstrated a link between chronotype and suicidal ideation [24], underscoring the impact of sleep patterns on mental health. Chronotypes vary with age, and evening-type preferences are more common during adolescence than morning-type preferences. However, adolescents have classes and engage in physical activities early in the morning, which leads to a lifestyle misaligned with their biological rhythms. This mismatch can lead to sleep deprivation and poor sleep quality. Evening-type individuals also typically exhibit higher levels of anxiety and depressive symptoms, along with a greater tendency for suicidal ideation [14]. Adequate sleep during the growth period positively affects emotional regulation and cognitive development, and contributes to both physical and mental health recovery [24]. Therefore, educational interventions that highlight the relationship between sleep patterns, chronotype, and depressive symptoms may help adolescents adjust their bedtimes and gradually shift from evening chronotypes to healthier sleep habits.

In our study, a high level of perceived health status was associated with a reduced risk of depressive symptoms. This finding aligns with Kim's study [25], which indicated that adolescents with a low perceived health status are more likely to experience depression than those with a high perceived health status. Adolescents tend to perceive their health not only in terms of physical well-being, but also in relation to social and emotional factors [37]. To reduce depressive symptoms by enhancing their perception of subjective health, it is important to help them understand that their health is also grounded in objective physical conditions. Furthermore, because peer relationships play a key role in adolescents' emotional well-being [3], interventions that foster positive peer interactions and attitudes are also essential.

When adolescents experience depression and attempt to cope by engaging in unhealthy behaviors (e.g., alcohol use, smoking, or drug misuse), they employ maladaptive coping strategies that can exacerbate emotional distress. In contrast, utilizing positive coping techniques such as modeling, communication training, and seeking professional help can alleviate negative emotions and lead to improved mental health outcomes [38]. The results of this study show that unhealthy behaviors such as drinking, smoking, and habitual drug use significantly increase the risk of depressive symptoms. This finding aligns with previous research involving middle- and high-school students, which indicated a correlation between depression and both alcohol consumption and smoking [6]. Additionally, this finding is consistent with results showing that adolescents with depressive symptoms had more drug use experiences than those without such symptoms [29]. Adolescent depressive symptoms tend to lead to increased alcohol consumption, smoking, and drug use, creating a cyclical structure that negatively affects physical and mental health [39]. Furthermore, physical and mental health status during adolescence significantly influences health outcomes in adulthood, with health behaviors established during this period often continuing into adulthood [40]. Therefore, it is essential to provide education and promote positive coping strategies rather than relying on health-risk behaviors to prevent adolescent depressive symptoms.

In this study, the mental health-related variables of anxiety, stress, and loneliness were found to significantly increase the risk for depressive symptoms. This aligns with previous research indicating that anxiety influences depression [2] and that higher perceptions of stress are associated with increased levels of depression [18]. Moreover, as the loneliness experience score increased, the risk of depressive symptoms also increased. This finding is consistent with previous research indicating that the prolonged feelings of loneliness resulting from the continuous school closures and social distancing measures during the COVID-19 pandemic have heightened the risk of depression among various mental health issues [41]. Given that anxiety, stress, and loneliness are risk factors for depressive symptoms, it is essential to develop targeted programs that address these domains. Evidence indicates that regular physical activity and strength training effectively reduce anxiety, depressive symptoms, and stress [42], while social interactions help alleviate loneliness [43]. Thus, physical activity programs incorporating social engagement may serve as effective strategies to promote adolescent mental health.

In addition, it is important to provide students with structured opportunities and sufficient time for positive stress management. Afterschool programs designed to reduce academic competition–related stress can play a crucial role, as psychological burdens and stress affect adolescents across all levels of academic performance [32]. Comprehensive efforts are therefore required to understand and mitigate academic stress in this population. Specifically, it is crucial to inform students that when they encounter overwhelming stress or feel depressed, they can seek solutions through support from those around them. To facilitate this, education on effective stress relief methods should be conducted for individuals or groups while promoting the formation of natural peer support and positive feedback. In the case of anxiety and loneliness, understanding and support from close individuals can help prevent the seriousness of these issues. Adolescents often find it challenging to recognize their own emotions and changes, and many do not know how to seek help from others [32]. Therefore, active assistance from nearby adults who can identify changes in adolescents is crucial. Additionally, there is a need for processes that help adolescents learn and practice methods for managing anxiety through counseling. For at-risk groups, it is important to provide support by utilizing youth counseling centers staffed with skilled counselors, ensuring systematic and in-depth counseling from the early stages of symptoms until their resolution [2]. For at-risk groups, it is important to strengthen the roles of parents, homeroom teachers, school health teachers, and other individuals in the community as active supporters and counselors for students' concerns [32].

According to Park [42], regular physical activity and strength training can serve as effective strategies for improving mental health by alleviating anxiety, depression, and stress among adolescents. However, the level of physical activity participation among Korean adolescents generally falls short of the recommendations of the World Health Organization [42]. The study indicated that, among men students, high-intensity physical activities and strength training were associated with significant reductions in anxiety disorders, whereas women students exhibited notable relationships with moderate-intensity physical activities. Moreover, given that most adolescents engage in physical activities through team sports, the social interactions that occur during these activities can also help reduce feelings of loneliness [43]. Building on this, developing and implementing physical activity programs aimed at enhancing adolescent mental health could not only have a positive impact on their mental well-being but also contribute to their overall health and fitness.

Active government policies, such as drug and alcohol awareness campaigns and preventive education, have been effective in reducing alcohol consumption and substance use [44]. School-based interventions have been able to decrease drinking behaviors [45,46], smoking, and drug use among adolescents [46,47]. Moreover, adolescents who receive more health education from school health teachers exhibit lower rates of smoking and alcohol use than their peers who do not receive such education [45]. Therefore, in conjunction with government-led awareness campaigns and preventive education centered on alcohol, tobacco, and drugs, the time allocated to health education within schools should be expanded, and a variety of health education programs to promote and maintain desirable health behaviors among adolescents should be implemented.

Our study has several limitations. First, the cross-sectional design precludes establishing causal relationships between subjective sleep patterns and self-reported depressive symptoms. Second, all variables were derived from self-reports in the KYRBS database [7], which may be subject to recall bias and underreporting of sensitive information. Third, depressive symptoms were assessed using a brief screening questionnaire rather than standardized clinical diagnostic criteria. Fourth, sleep data reflected only the previous week [7], which may not accurately represent typical sleep patterns across the academic year. Fifth, we were unable to include objective sleep measures or comprehensive assessments of sleep quality. Finally, cultural factors specific to Korean adolescents, such as academic achievement pressure [32], may limit the generalizability of our findings to other populations. Nevertheless, this study is valuable in that it provides nationally representative data on the relationships between depressive symptoms, sleep patterns, and chronotype among Korean adolescents. These findings indicate that circadian disruption—such as delayed sleep onset and curtailed sleep—likely disturbs the secretory patterns of serotonin, norepinephrine, dopamine, and melatonin, thereby increasing the risk of depressive symptoms in adolescents. This mechanistic link supports integrating circadian and sleep screening and education into basic nursing science curricula.

## CONCLUSIONS

An increase in sleep duration was associated with a reduced risk of belonging to the depression symptoms group among adolescents, indicating that sleep factors related to bedtime, wake-up time, and chronotype are associated with depressive symptoms. To reduce depression symptoms in adolescents, it is essential to promote earlier bedtimes, later wake-up times, and improved evening chronotypes, thereby extending total sleep duration. To achieve this, health education and campaigns focused on raising awareness of sleep and depression are necessary. Furthermore, preventing drinking, smoking, and habitual drug use during adolescence can foster the development of healthy lifestyle habits that help prevent depression symptoms. In addition, it is vital to implement integrated mental health promotion programs that address and prevent anxiety, stress, and loneliness, which increase the risk of depressive symptoms.

## Figures and Tables

**Table 1.** Weighted Prevalence of Depressive Symptoms, Sleep Patterns, and Chronotype in Adolescents in Korea (N = 46,987)

Variables	Categories	Total
		n (%) or M ± SE
Depressive symptoms	Yes	11,967 (25.3)
	No	35,020 (74.7)
Bedtime	20:00~21:59	602 (1.1)
	22:00~23:59	10,743 (21.0)
	00:00~00:59	10,543 (22.1)
	01:00~01:59	12,744 (27.9)
	02:00~02:59	8,926 (20.1)
	03:00~03:59	3429 (7.8)
Wake-up time	04:00~04:59	137 (0.3)
	05:00~05:59	1,443 (2.6)
	06:00~06:59	13,072 (27.1)
	07:00~07:59	28,613 (61.2)
	08:00~08:59	3,722 (8.8)
Sleep duration (hours)		6.24 ± 0.01
	< 4	1,631 (3.5)
	≥ 4 and < 5	5,708 (12.6)
	≥ 5 and < 6	10,964 (24.3)
	≥ 6 and < 7	12,078 (25.9)
	≥ 7 and < 8	9,678 (19.9)
	≥ 8 and < 10	6,728 (13.4)
	≥ 10	200 (0.4)
Chronotype MSF_SC_		5.39 ± 0.01

M = Mean; SE = Standard error; MSF_SC_ = Mid-sleep time on free days corrected for sleep debt accumulated over school days.

**Table 2.** Comparison of General Characteristics and Health-related Characteristics by Depressive Symptoms in Adolescents (N = 46,987)

Variables	Categories	Total	Depressive symptoms (n = 11,967)	No depressive symptoms (n = 35,020)	χ² or t	*p*
General characteristics						
Age (years)		15.18 ± 0.03	15.16 ± 0.03	15.18 ± 0.03	0.82	.411
Sex	Men	23,780 (51.6)	4,957 (42.5)	18,823 (54.7)	451.10	< .001
	Women	23,207 (48.4)	7,010 (57.5)	16,197 (45.3)		
School year	Middle school	25,025 (50.5)	6,454 (51.3)	18,571 (50.2)	2.10	.146
	High school	21,962 (49.5)	5,513 (48.7)	16,449 (49.8)		
Living with family	Yes	44,909 (96.3)	11,306 (95.2)	33,603 (96.6)	40.52	< .001
	No	2,078 (3.7)	661 (4.8)	1,417 (3.4)		
Household income	High	5,490 (12.1)	1,315 (11.3)	4,175 (12.4)	140.41	< .001
	Middle	31,971 (86.2)	10,277 (85.7)	30,380 (86.3)		
	Low	840 (1.7)	375 (3.0)	465 (1.3)		
Academic performance	High	6,090 (13.0)	1,264 (10.6)	4,826 (13.8)	200.17	< .001
	Middle	36,835 (78.4)	9,319 (77.9)	27,516 (78.6)		
	Low	4,062 (8.6)	1,384 (11.5)	2,678 (7.6)		
Perceived health status		3.75 ± 0.01	3.49 ± 0.01	3.84 ± 0.01	31.81	< .001
BMI	Low	3,928 (8.5)	1,046 (8.9)	2,882 (8.4)	3.72	.155
	Normal	33,043 (70.6)	8,438 (70.6)	24,605 (70.6)		
	Obese	10,016 (20.9)	2,483 (20.5)	7,533 (21.0)		
Health behavior						
Current drinking	Yes	4,898 (10.5)	1,841 (15.3)	3,057 (8.9)	289.83	< .001
	No	42,089 (89.5)	10,126 (84.7)	31,963 (91.1)		
Current smoking	Yes	1,790 (3.8)	799 (6.8)	991 (2.8)	259.46	< .001
	No	45,197 (96.2)	11,168 (93.2)	34,029 (97.2)		
Moderate physical activity	Yes	8,292 (17.1)	2,140 (17.4)	6,152 (17.0)	0.73	.391
	No	38,695 (82.9)	9,827 (82.6)	28,868 (83.0)		
Vigorous physical activity	Yes	19,776 (41.2)	4,980 (40.8)	14,796 (41.3)	0.78	.376
	No	27,211 (58.8)	6,987 (59.2)	20,224 (58.7)		
Experience of habitual drug use	Yes	606 (1.4)	358 (3.2)	248 (0.7)	261.73	< .001
	No	46,381 (98.6)	11,609 (96.8)	34,772 (99.3)		
Mental health variables						
Awareness of stress		3.25 ± 0.01	3.79 ± 0.01	3.07 ± 0.01	−70.80	< .001
Experience of loneliness		2.58 ± 0.01	3.31 ± 0.01	2.34 ± 0.01	−88.33	< .001
Anxiety	Yes	5,663 (12.1)	3,642 (30.5)	2,021 (5.9)	6115.38	< .001
	No	41,324 (87.9)	8,325 (69.5)	32,999 (94.1)		

Values are presented as the mean ± standard deviation or n (%). BMI = Body mass index.

**Table 3.** Comparison of Sleep Patterns and Chronotype by Depressive Symptoms in Adolescent (N = 46,987)

Variables	Categories	Depressive symptoms (n = 11,967)	No depressive symptoms (n = 35,020)	χ² or t	*p*
Bedtime	20:00~21:59	144 (24.2)	458 (75.8)	388.93	< .001
	22:00~23:59	2,199 (20.3)	8,544 (79.7)		
	00:00~00:59	2,444 (22.8)	8,099 (77.2)		
	01:00~01:59	3,285 (25.3)	9,459 (74.7)		
	02:00~02:59	2,672 (29.6)	6,254 (70.4)		
	03:00~03:59	1,223 (35.2)	2,206 (64.8)		
Wake-up time	04:00~04:59	49 (36.5)	88 (63.5)	73.30	< .001
	05:00~05:59	449 (31.7)	994 (68.3)		
	06:00~06:59	3,574 (27.3)	9,498 (72.7)		
	07:00~07:59	6,917 (24.1)	21,696 (75.9)		
	08:00~08:59	978 (25.7)	2,744 (74.3)		
Sleep duration (hours)		5.59 ± 0.02	6.32 ± 0.01	21.11	< .001
	< 4	629 (38.7)	1,002 (61.3)	452.87	< .001
	≥ 4 and < 5	1,869 (32.3)	3,839 (67.7)		
	≥ 5 and < 6	3,050 (27.6)	7,914 (72.4)		
	≥ 6 and < 7	2,968 (24.3)	9,110 (75.7)		
	≥ 7 and < 8	2,088 (21.2)	7,590 (78.8)		
	≥ 8 and < 10	1,315 (19.5)	5,413 (80.5)		
	≥ 10	48 (21.7)	152 (78.3)		
Chronotype MSF_SC_		5.49 ± 0.03	5.36 ± 0.02	−4.72	< .001

Values are presented as the mean ± standard deviation or n (%). MSF_SC_ = Mid-sleep time on free days corrected for sleep debt accumulated over school days.

**Table 4.** Weighted Multivariate Logistic Regression Analysis of Factors Associated with Depressive Symptoms in Adolescents (N = 46,987)

Variables	Categories	Adjusted OR (95% CI)	*p*
Sex	Men	1.00	
	Women	1.15 (1.09~1.22)	< .001
Living with family	Yes	1.00	
	No	1.19 (1.05~1.35)	< .001
Household income	High	1.00	
	Middle	0.81 (0.74~0.89)	< .001
	Low	1.17 (0.95~1.45)	.145
Academic performance	High	1.00	
	Middle	1.30 (1.20~1.41)	< .001
	Low	1.48 (1.31~1.67)	< .001
Current drinking	No	1.00	
	Yes	1.35 (1.24~1.48)	< .001
Current smoking	No	1.00	
	Yes	1.72 (1.50~1.96)	< .001
Experience of habitual drug use	No	1.00	
	Yes	1.98 (1.57~2.49)	< .001
Perceived health status		0.96 (0.93~0.99)	.024
Anxiety	No	1.00	
	Yes	2.28 (2.11~2.46)	< .001
Awareness of stress		1.72 (1.66~1.79)	< .001
Experience of loneliness		2.00 (1.94~2.07)	< .001
Sleep duration (hours)		0.98 (0.96~0.99)	.021

OR = Odds ratio; CI = Confidence interval.

## Data Availability

The data that supports the findings of this study are available from the corresponding author upon reasonable request.
